# Pathology and molecular characterization of *Leucocytozoon caulleryi* from backyard chickens in Khon Kaen Province, Thailand

**DOI:** 10.14202/vetworld.2021.2634-2639

**Published:** 2021-10-09

**Authors:** Tawatchai Pohuang, Suphattra Jittimanee, Sucheeva Junnu

**Affiliations:** 1 Division of Livestock Medicine, Faculty of Veterinary Medicine, Khon Kaen University, Khon Kaen 40002, Thailand; 2 Research Group for Emerging and Re-emerging Infectious Diseases in Animals and Zoonotic Diseases, Faculty of Veterinary Medicine, Khon Kaen University, Khon Kaen 40002, Thailand; 3 Division of Pathobiology, Faculty of Veterinary Medicine, Khon Kaen University, Khon Kaen 40002, Thailand.

**Keywords:** chickens, cytochrome c oxidase subunit I gene, leucocytozoonosis, megaloschizonts

## Abstract

**Aim:**

The aim of this study was to characterize *Leucocytozoon caulleryi* from backyard chickens in Khon Kaen Province, Thailand.

**Materials and Methods:**

Tissue samples were collected from backyard chickens suspected to have leucocytozoonosis and subjected to histopathology examination. The BLAST Tool at NCBI GenBank (Basic Local Alignment Research Tools) (http://www.ncbi.nlm.nih.gov/BLAST) was used to identify the nucleotide sequence of the partial cytochrome c oxidase subunit I (*cox I)* gene. A Phylogenetic tree for analysis of *L. caulleryi* was constructed by using MEGA 7.0 software (https://www.megasoftware.net/).

**Results:**

The necropsy results revealed the subcutaneous hemorrhages of pectoral muscles, multifocal hemorrhages of the thymus and pectoral muscles, hemorrhage of the proventriculus and peritoneal cavity, and megaloschizonts of the pancreas and intestine, including subcapsular hemorrhages of the liver. Microscopic examination revealed numerous megaloschizonts of *Leucocytozoon* spp. in the pectoral muscles, intestine, pancreas, and thymus. Molecular analysis of the partial *cox I* gene showed that the causal agent was closely related to *L. caulleryi* reported in Japan.

**Conclusion:**

From these results, *L. caulleryi* was determined to be the causal agent of leucocytozoonosis and was closely associated with *L. caulleryi* reported in Japan.

## Introduction

Leucocytozoonosis is an avian hemoparasitic disease caused by parasitic protozoa of the genus *Leucocytozoon* in the phylum *Apicomplexa* (class Aconoidasida, subclass Haemosporidiasina, and order Haemospororida) and the family Leucocytozoidae. *Leucocytozoon caulleryi* has been reported in chickens in southern and eastern Asia [1-3]. Chickens become infected with *Leucocytozoon* spp. through *Culicoides* spp. (*Culicoides arakawa*) and *Simulium* spp. [4]. In Thailand, the first leucocytozoonosis case was reported in 1954 [5].

Infected birds exhibit acute clinical signs such as internal bleeding, anemia, lethargic, diarrhea, pallor, and experience mortality and reduced egg production [6,7]. The schizogonies and sporozoites stages are transmitted through the salivary glands of an insect vector, and developed to the first generation of schizonts in various organs following invasion of the vascular endothelium. The second generation of megaloschizonts was identified using histopathological procedures. At the gametogony stage, merozoites are released from megaloschizonts and transformed into gametocytes in erythrocytes [8]. Therefore, leucocytozoonosis can be diagnosed by microscopic examination, and identified from stained blood or infected tissue sections, or using molecular assays [9,10]. In general, infection with *Leucocytozoon* spp. is identified using polymerase chain reaction (PCR), which is more sensitive than microscopic examination because the DNA of the parasite can be detected even when gametocytes are not found in thin blood smears [11]. There are no drugs available to treat avian leucocytozoonosis. As previously reported, treatment with pyrimethamine combined with sulfadimethoxine was found to be partially effective, while primaquine was active against *Leucocytozoon* spp. gametocytes [11]. Vaccination was carried out with the second generation schizonts and oil-adjuvant recombinant R7 that presented effective results in laboratory and field trials [12,13].

The *L. caulleryi* mitochondrial genome encodes three protein-coding genes, cytochrome b (*cyt b*), cytochrome c oxidase subunits I (*cox I*) and III (*cox III*), and large subunit rRNA genes [14]. The *cyt b* and *cox I* genes have been extensively used for the detection of avian *L. caulleryi* [15,16]. The *cox I* gene is a highly conserved mitochondrially encoded protein and so is frequently used for species determination and phylogenetical analysis studies [17].

The objectives of this research were to identify the species of *Leucocytozoon* and study the phylogeny of *Leucocytozoon calleryi* from backyard chickens in Thailand. Gross and microscopic examination and molecular techniques were used to characterize chicken leucocytozoonosis cases.

## Materials and Methods

### Ethical approval

The study was approved by the Institutional Animal Care and Use Committee of Khon Kaen University, based on the Ethics of Animal Experimentation of National Research Council of Thailand, No: IACUC-KKU-55/64.

### Study period, location, and sampling

In June 2018, five 12-to-18-day-old carcasses of backyard chickens from Khon Kaen Province in Thailand were subjected for necropsy at the Veterinary Diagnostic Laboratory, Faculty of Veterinary Medicine, Khon Kaen University, Khon Kaen, Thailand. These birds were submitted from a flock with a history of depression, subcutaneous hemorrhages, and mortality. The mortality was 100% within 1 week.

### Gross histopathology

Tissue samples from lung, heart, liver, thymus, pectoral muscle, proventriculus, gizzard, intestine, and pancreas were collected from infected chicken carcasses. The tissues were fixed with 10% neutral buffered formalin solution and embedded in paraffin sections that were stained with hematoxylin and eosin for histopathological examination.

### DNA extraction and PCR assays

Genomic DNA was extracted from tissue of the infected chicken using NucleoSpin Tissue, Mini kits for DNA (Macherey-Nagel, Düren, Germany), following the manufacturer’s instructions. PCR reactions were performed with a pair of primers, as described previously, to amplify the 588 bp mitochondrial *cox I* gene [18]. The PCR reaction was carried out in a DNA thermal cycler (Major Cycler, Major Science, Taiwan) using DreamTaq Green PCR Master Mix (2X) (Thermo Fisher Scientific, Waltham, MA, USA) according to the manufacturer’s instructions. Briefly, 3 μL of the DNA template was added to 22 μL of PCR reaction mixture consisting of 12.5 μL of DreamTaq Green PCR Master Mix solution (Thermo Fisher Scientific, Waltham, MA, USA), 0.5 μL each of 10 μM forward and reverse primers, and 8.5 μL of nuclease-free water. Thermal cycling was performed with an optimized profile of initial denaturation at 95°C for 5 min, followed by 40 cycles at 95°C for 30 s, 55°C for 30 s, and 68°C for 60 s, and a final extension at 68°C for 10 min. The PCR products were analyzed using electrophoresis in 1.2% agarose gels containing RedSafe Nucleic Acid Staining Solution (JH Science, Kirkland, WA, USA). The gels were run at 100 volts for 30 min and visualized using ultraviolet light in a Gel Doc™ XR+ Gel Documentation System (Bio-Rad, Hercules, CA, USA). The PCR products of the PCR-positive samples were reamplified for gel purification using NucleoSpin Gel and PCR Clean-up kit (Macherey-Nagel, Düren, Germany) following the manufacturer’s instructions, and subjected to direct sequencing by First BASE Laboratories Sdn Bhd, Seri Kembangan, Selangor, Malaysia.

### Phylogenetic analysis

The nucleotide sequences were analyzed for sequence identity using the BLAST Tool at NCBI GenBank (Basic Local Alignment Search Tools) (www.ncbi.nlm.nih.gov/BLAST) and assembled using BioEdit software version 7.1.11 (https://bioedit.software.informer.com/7.1/). The sequences were deposited in the GenBank database using the BankIt tool (www.ncbi.nlm.nih.gov/WebSub/?tool=genbank). The GenBank accession number MK903018 was assigned to KKUTH. Phylogenetic tree analysis was carried out using the MEGA 7.0 program (https://www.megasoftware.net/) with the neighbor-joining method. Statistical analyzes involved 1000 bootstrap replicates. The phylogenetic trees were constructed from reference strains of GenBank accession numbers AB250690.1, KP025674.1, AB250415.1, AB302215.1, AB299369.1, KT162004.1, and FJ168563.1.

## Results

### Gross histopathology

**Figure-1:**
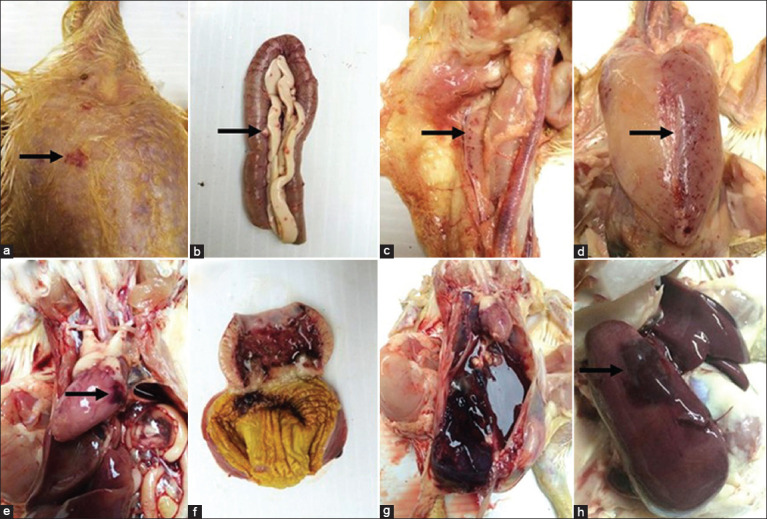
Gross lesions of *Leucocytozoon caulleryi* infecting backyard chickens. (a) Subcutaneous hemorrhage in pectoral, (b) multifocal hemorrhage and megaloschizont in the pancreas and intestine, (c) thymus, (d) pectoral muscles, (e) petechial hemorrhage in epicardium, (f) hemorrhage in proventriculus and gizzard, (g) hemorrhage in the peritoneal cavity, (h) liver lobe was enlarged and had a subcapsular hemorrhagic region (arrows).

**Figure-2:**
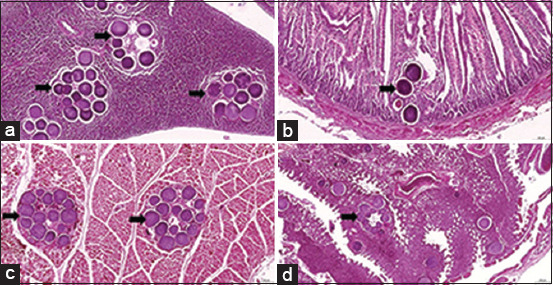
Microscopic appearance of *Leucocytozoon caulleryi* infection in chickens. Histopathological findings in the pancreas, intestine, muscle, and lung from the case of chicken Leucocytozoonosis in backyard chicken. A, B, C, and D: Hematoxylin and eosin staining showed the numerous megaloschizont (arrow) of *L. caulleryi* in pancreas, intestine, muscle, and lung, respectively.

Gross pathology of the birds showed subcutaneous hemorrhages of the wings and legs, including multifocal hemorrhages in the pectoral and thigh muscles, thymus and bursa of Fabricius, with friable hepatic lobes and splenomegaly. Two birds were found to have subcapsular liver hemorrhages. Other lesions included petechiae of the lung, heart, proventriculus, gizzard, pancreas, liver, and kidney in some birds (Figure-1). The pectoral muscles, intestine, pancreas, and thymus carried numerous *Leucocytozoon* megaloschizonts and merozoites (Figure-2).

### Phylogenetic analysis

**Figure-3:**
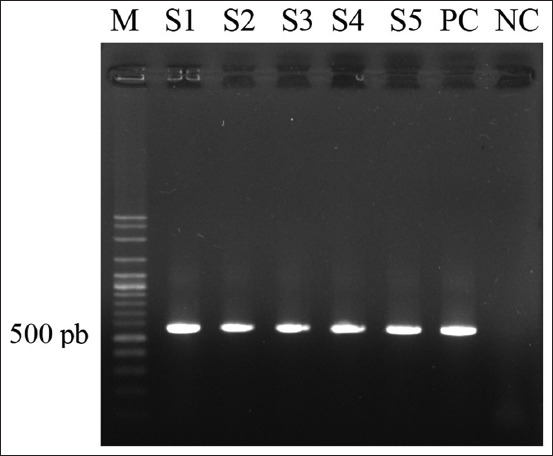
Polymerase chain reaction electrophoresis gel demonstrating *Leucocytozoon caulleryi cox I* gene amplification with *Cox I*-F1 and *Cox I*-R1. M. 100-bp DNA marker ladder, Lanes: 1. Sample1; 2. Sample2; 3. Sample3; 4. Sample4; 5. Sample5; NC, negative control; PC, positive control.

**Figure-4:**
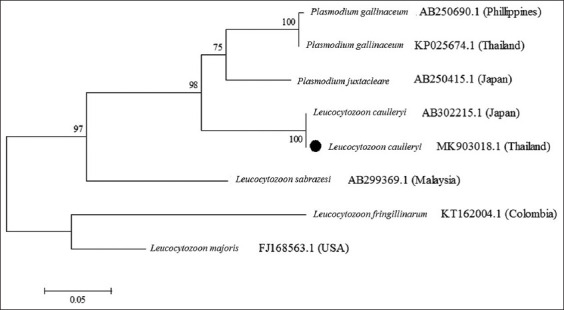
Neighbor-joining phylogenetic tree based on the partial *cox I* gene (588-bp) of *Leucocytozoon caulleryi* in backyard chickens in Khon Kaen Province (black circle). *Plasmodium juxtanucleare* and *Plasmodium gallinaceum* as an out group and *Leucocytozoon* spp. from other species was taken from GenBank. Four strains are indicated that the *Leucocytozoon* species name, the accession number and the country where the pathogen was reported. Bootstrap values are listed as percentages after 1000 replications.

A total of 588 bp of PCR products were successfully amplified (Figure-3) and all five DNA sequences were identical to the *cox I* gene of *L. caulleryi*. The obtained sequence was deposited in GenBank with the accession number MK903018. Sequence analysis showed 100% nucleotide sequence homology with the reference strain, and complete identity with the *L. caulleryi* mitochondrial DNA genome from Japan (GenBank accession no. AB302215.1) (Figure-4).

## Discussion

The objective of this study was to characterize *L. caulleryi* infecting backyard chickens using gross histopathology and molecular techniques. Gross lesions were found in various organs examined, including subcutaneous hemorrhages of pectoral muscles, legs and wings, subcapsular liver hemorrhage, and hemorrhages in pectoral and thigh muscles and the thymus. These lesions were similar to those reported from a previous study of broiler breeders in South Korea, which found subcutaneous hemorrhages in the legs and wings, thigh and pectoral muscles, thymus, heart, pancreas, and kidneys [2]. Leucocytozoonosis cases in South Korea present with gross lesions of the friable hepatic lobes, splenomegaly, and degenerated ovaries and oviducts in layer chickens from commercial farms [19]. These lesions cause high mortality, causing economic losses to the poultry industry.

*L. caulleryi* frequently causes lethal hemorrhagic disease in chickens [6], and anemia due to the destruction of gametocytes, erythrocytes, and the vascular endothelium. Five birds in this report died with clinical signs, including depression and subcutaneous hemorrhage. The clinical signs of protozoal disease and mortality result from anemia produced by antierythrocytic factors, as large numbers of gametocytes block pulmonary capillaries or parasites invade the vessels and vascular endothelium of important organs such as the brain and heart. The parasites form megaloschizonts that block the vessels, resulting in multifocal necrosis [8]. *L. caulleryi* infection in layer chickens causes mortality and reduced egg production [20,21]. In Thailand, there have been reports of the prevalence of blood parasites, including *L. caulleryi*, in backyard chickens and fighting cocks [22,23].

Microscopic histopathological examination is used to provide definitive diagnoses of *L. caulleryi* infections in chickens [24,25]. Histopathological studies of *L. caulleryi* in this study revealed numerous megaloschizonts in organs such as the pancreas, intestine, lung, and pectoral muscle. Petechiae were found in muscle tissue and megalozchizonts in spleen, liver, lung, and heart [26].

Molecular analysis of a *cox I* gene indicated that *L. caulleryi* in this study was phylogenetically identical to those previously reported in Japan, where avian leucocytozoonosis is endemic [3,14]. In this study, DNA was directly extracted from the tissue samples. Other studies have separated the gametocytes of *L. caulleryi* from whole blood using flow cytometry, and the gametocytes might be used for further genetic analyzes [27]. These results indicated that molecular techniques were useful for the diagnosis of diseases and the development of intervention strategies for avian leucocytozoonosis.

The arthropod vectors and their population density were considered for disease control and the eradication in vectors of backyard and free-range chickens. *C. arakawa* has been identified in chickens positive for *Leucocytozoon* spp. reared in open houses [5]. The prevalence of this protozoal disease frequently increases during the spring season due to arthropod vectors such as black flies (*Simulium* spp.) and biting midges (*Culicoides* spp.) [28]. Study of *Culicoides* spp. have been reported in several countries in Asia with climates suitable for the survival and reproduction of insect vectors [29]. The prevalence of leucocytozoonosis in chickens increases in late summer or early in the rainy season because this period facilitates *Culicoides arakawae* distribution throughout Asian countries. In Thailand, the prevalence of infection with blood parasites in backyard chickens was highest in the summer season, even higher than the rates of infection in the rainy season [22]. *Simulium* *asakoae* complex and *Simulium chumpornense* have been reported to be possible vectors of *Leucocytozoon* in poultry and wild birds in Thailand [29]. Thus, the occurrence of avian leucocytozoonosis is related to the distribution of *C. arakawae* [5], and disease control in chickens requires the elimination of insect vectors from the environment [30].

## Conclusion

Chickens that died suddenly showed clinical signs of depression and pallor, and gross lesions, including subcutaneous hemorrhages, multifocal hemorrhages of various organs. *L. caulleryi* was determined to be the causal agent and was very similar to the strain reported in Japan. Histopathological examination and molecular analysis are valuable tools for the detailed investigation of avian leucocytozoonosis.

## Authors’ Contributions

SJ, TP, and SJ: Designed the work. SJ and TP: Collected the samples. SJ: Carried out the histopathology laboratory experiment and drafted the manuscript. All authors read and approved the final manuscript.
